# Supplementary figures and images for: An amalgamation of YOLOv4 and XGBoost for next-gen smart traffic management system
Source: PeerJ Comput Sci. 2021 Jun 18;7:e586. doi: 10.7717/peerj-cs.586 (PMC8237335; doi:10.7717/peerj-cs.586)

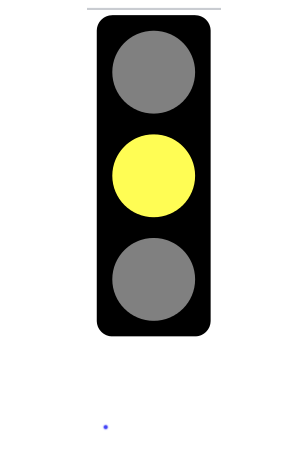

Supplement: Supplemental Information 1 — The YOLO object detection analysis and prediction models are in the Jupyter-Notebook file. The main script which starts the system and other files referenced with the main script is available in the Python file and the whole system is implemented in python programming language. The pretrained model of YOLOv4 is available at GitHub: https://github.com/AlexeyAB/darknet/wiki/YOLOv4-model-zoo. [file peerj-cs-07-586-s001.zip › System_Implementation/traffic_lights/yellow.png]

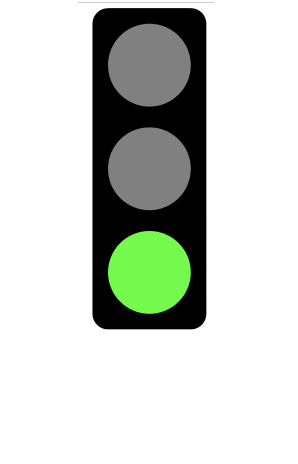

Supplement: Supplemental Information 1 — The YOLO object detection analysis and prediction models are in the Jupyter-Notebook file. The main script which starts the system and other files referenced with the main script is available in the Python file and the whole system is implemented in python programming language. The pretrained model of YOLOv4 is available at GitHub: https://github.com/AlexeyAB/darknet/wiki/YOLOv4-model-zoo. [file peerj-cs-07-586-s001.zip › System_Implementation/traffic_lights/green.png]

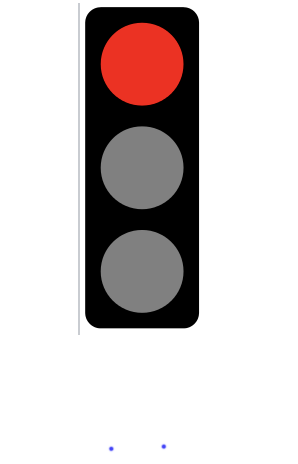

Supplement: Supplemental Information 1 — The YOLO object detection analysis and prediction models are in the Jupyter-Notebook file. The main script which starts the system and other files referenced with the main script is available in the Python file and the whole system is implemented in python programming language. The pretrained model of YOLOv4 is available at GitHub: https://github.com/AlexeyAB/darknet/wiki/YOLOv4-model-zoo. [file peerj-cs-07-586-s001.zip › System_Implementation/traffic_lights/red.png]
